# US Clinicians’ Experiences and Perspectives on Resource Limitation and Patient Care During the COVID-19 Pandemic

**DOI:** 10.1001/jamanetworkopen.2020.27315

**Published:** 2020-11-06

**Authors:** Catherine R. Butler, Susan P. Y. Wong, Aaron G. Wightman, Ann M. O’Hare

**Affiliations:** 1 Kidney Research Institute, Division of Nephrology, Department of Medicine, University of Washington, Seattle; 2 Nephrology Section, Hospital and Specialty Medicine and Seattle-Denver Health Services Research and Development Center of Innovation, Veterans Affairs Puget Sound Health Care System, Seattle, Washington; 3 Department of Pediatrics, University of Washington School of Medicine, Seattle; 4 Treuman Katz Center for Pediatric Bioethics, Seattle Children’s Hospital, Seattle, Washington

## Abstract

**Importance:**

Little is known about how US clinicians have responded to resource limitation during the coronavirus disease 2019 (COVID-19) pandemic.

**Objective:**

To describe the perspectives and experiences of clinicians involved in institutional planning for resource limitation and/or patient care during the pandemic.

**Design, Setting, and Participants:**

This qualitative study used inductive thematic analysis of semistructured interviews conducted in April and May 2020 with a national group of clinicians (eg, intensivists, nephrologists, nurses) involved in institutional planning and/or clinical care during the COVID-19 pandemic across the United States.

**Main Outcomes and Measures:**

Emergent themes describing clinicians’ experience providing care in settings of resource limitation.

**Results:**

The 61 participants (mean [SD] age, 46 [11] years; 38 [63%] women) included in this study were practicing in 15 US states and were more heavily sampled from areas with the highest rates of COVID-19 infection at the time of interviews (ie, Seattle, New York City, New Orleans). Most participants were White individuals (39 [65%]), were attending physicians (45 [75%]), and were practicing in large academic centers (≥300 beds, 51 [85%]; academic centers, 46 [77%]). Three overlapping and interrelated themes emerged from qualitative analysis, as follows: (1) planning for crisis capacity, (2) adapting to resource limitation, and (3) multiple unprecedented barriers to care delivery. Clinician leaders worked within their institutions to plan a systematic approach for fair allocation of limited resources in crisis settings so that frontline clinicians would not have to make rationing decisions at the bedside. However, even before a declaration of crisis capacity, clinicians encountered varied and sometimes unanticipated forms of resource limitation that could compromise care, require that they make difficult allocation decisions, and contribute to moral distress. Furthermore, unprecedented challenges to caring for patients during the pandemic, including the need to limit in-person interactions, the rapid pace of change, and the dearth of scientific evidence, added to the challenges of caring for patients and communicating with families.

**Conclusions and Relevance:**

The findings of this qualitative study highlighted the complexity of providing high-quality care for patients during the COVID-19 pandemic. Expanding the scope of institutional planning to address resource limitation challenges that can arise long before declarations of crisis capacity may help to support frontline clinicians, promote equity, and optimize care as the pandemic evolves.

## Key Points

**Question:**

How have US clinicians planned for and responded to resource limitation during the coronavirus disease 2019 pandemic?

**Findings:**

This qualitative study included interviews with 61 clinicians across the United States. While institutions planned for an explicit and systematic approach to resource allocation in crisis settings, this approach did not address many challenges encountered by frontline clinicians, leaving them to struggle with what constituted acceptable standards of care and to make difficult allocation decisions.

**Meaning:**

The findings of this study suggest that expanding the scope of institutional planning to address a broader spectrum of resource limitation may help to support clinicians, promote equity, and optimize care during the pandemic.

## Teaser

This qualitative study describes the perspectives and experiences of clinicians involved in institutional planning for resource limitation and/or patient care during the coronavirus disease 2019 (COVID-19) pandemic.

## Introduction

Since the first US case of coronavirus disease 2019 (COVID-19) was diagnosed in mid-January 2020,^1^ the pandemic has completely transformed health care delivery in this country. Early reports from frontline clinicians in global epicenters describing extreme shortages and bedside rationing of ventilators and intensive care unit (ICU) beds^2^ prompted a national conversation about how to respond to similar challenges in the United States.^3^ Hospitals and health care systems drew on frameworks developed by the Institute of Medicine (IOM) and other national organizations to guide care in resource-limited emergency settings.^4,5,6,7^

Under the IOM’s framework, resource allocation is intentionally siloed from other aspects of clinical care to ensure a fair process and spare frontline clinicians from the responsibility of having to ration scarce resources at the bedside. The IOM recommends a phased adaptation to resource limitation. Institutions first shift from conventional to contingency capacity, in which resources are adapted, optimized, and redistributed to maintain a standard of care that is functionally equivalent to usual care. If resources become so limited that a functionally equivalent standard of care can no longer be sustained, institutions then shift to crisis capacity, and care is redirected to provide the greatest aggregate benefit to the population.^8^ Under crisis standards of care, a specialized triage team becomes responsible for rationing scarce resources and making decisions about which patients will and will not receive potentially life-saving treatments.

Although the IOM framework reflects lessons learned during earlier pandemics^9,10^ and has been iteratively refined through ethical analysis^8^ and community deliberation,^11,12^ there have been few opportunities to test the framework in real-world clinical settings. Reports from past pandemics^13,14^ and from early global epicenters of the COVID-19 pandemic,^15^ press reports,^16,17^ and perspectives published in the scientific literature^18,19^ describe some of the challenges faced by frontline clinicians. However, there is little empirical work describing the experiences and perspectives of US clinicians pertaining to resource limitation and clinical care during the COVID-19 pandemic.^20,21^ To address this knowledge gap, we conducted a qualitative analysis of interviews with US clinicians during the pandemic.

## Methods

### Participants

We recruited clinicians from across the United States who had cared for patients during the COVID-19 pandemic and/or had been involved in planning institutional responses to resource limitation. We used purposive sampling to select a group of participants with a variety of roles who were practicing in a range of different settings. We began by recruiting clinicians with direct experience planning for and/or practicing in settings of resource limitation (intensivists, nephrologists, and triage team members) at our own institution (the University of Washington). We then expanded recruitment to include other groups of clinicians (eg, trainees, palliative care specialists, nurse care coordinators) and those practicing in other parts of the country. We used a snowballing recruitment strategy in which we asked participants to identify colleagues with relevant experience who might be interested in participating in the study. We did not exclude participants who had collegiate relationships with members of the research team. Interviews were conducted between April 9, 2020, and May 26, 2020. The University of Washington institutional review board approved this study and authorized verbal in lieu of written consent. Verbal consent was obtained from all participants. We report details of our methods using the Consolidated Criteria for Reporting Qualitative Research (COREQ) reporting guideline (eTable 1 in the Supplement).^22^

### Data Collection

Participating clinicians completed one 30-minute to 60-minute audio-recorded interview with 1 of us (C.R.B., a White, female senior nephrology fellow trained in qualitative methodology and clinical bioethics). All but 1 interview, which included 2 participants at their request, were conducted 1-on-1, and 2 interviews were spread over 2 sittings. A semistructured interview guide (eTable 2 in the Supplement) was developed by 3 of us (C.R.B., A.M.O., and S.P.Y.W; A.M.O. and S.P.Y.W. are academic nephrologists and physician scientists with experience in qualitative methodology, geriatric nephrology, and palliative care) and included open-ended questions to elicit clinicians’ perspectives and experiences pertaining to clinical care, institutional policies, and resource limitation during the pandemic. The interview guide was iteratively refined by 1 of us (C.R.B.) with input from 2 of us (A.M.O. and S.P.Y.W.) to allow for elaboration of emerging themes. Interviews were recorded and transcribed verbatim. To protect confidentiality, participants were offered the opportunity to review their written transcripts to confirm accuracy and identify passages that they did not want published, but they were not invited to review draft or final versions of the article. Participants also completed an online survey with questions about their demographic characteristics and practice experience. The size of participants’ home hospital (or the hospital at which they volunteered, if that was the focus of the interview) was ascertained by online search.

### Statistical Analysis

We conducted an inductive thematic analysis^23^ of interview transcripts with the goal of discovering emergent themes describing clinicians’ perspectives, experiences, and practices pertaining to resource limitation during the COVID-19 pandemic. Two of us (C.R.B. and A.M.O.) independently reviewed and openly coded transcripts until reaching thematic saturation (ie, the point at which no new concepts were identified),^24,25^ which occurred after reviewing 30 interview transcripts. One of us (C.R.B.) coded all remaining transcripts for concurrence. Throughout the analysis, the 2 investigators iteratively reviewed codes, collapsed codes into groups with related meanings and relationships, and developed larger thematic categories, returning frequently to the transcripts to ensure that emergent themes were well-grounded in the data.^23,25^ All coauthors (including A.G.W., a pediatric nephrologist, physician scientist, and bioethicist) reviewed example quotations and themes and together developed the final thematic schema. We used Atlas.ti version 8 (Scientific Software Development GmbH) to organize and store text and codes.

## Results

We approached a total of 97 clinicians by email, of whom 75 (77%) agreed to participate and 22 (23%) declined or did not respond to our inquiry. Among those who agreed to participate, we purposively sampled 61 clinicians to participate in interviews from April 9, 2020, to May 26, 2020 (Table 1).^26^ All but 1 participant completed the online survey. The mean (SD) age of participants was 46 (11) years, and most were White (39 [65%]), were attending physicians (45 [75%]), and were practicing in large academic hospitals (≥300 beds, 51 [85%]; academic, 46 [77%]). Participants were practicing in 15 states across the United States and had primary affiliations with 29 different hospitals or clinics, with greater sampling of clinicians practicing in states with the highest rates of COVID-19 infection at the time of the study (eg, Seattle, New York City, New Orleans).

**Table 1.** Participant Characteristics

Characteristic	Participants (N = 60)^a^
Age, mean (SD), y	45.8 (11.1)
Gender	
Women	38 (63.3)
Men	22 (36.6)
Race	
Asian or South Asian	15 (25.0)
Black or African American	2 (3.3)
White	39 (65.0)
≥1 or other	3 (5.0)
Prefer not to say	1 (1.7)
Ethnicity	
Hispanic or Latino	1 (1.7)
Not Hispanic or Latino	58 (96.7)
Prefer not to say	1 (1.7)
Type of institution	
Academic	46 (76.7)
Private	9 (15.0)
Other	5 (8.3)
Clinical site^b^	
Clinic or outpatient	38 (63.3)
Inpatient acute care	41 (68.3)
Inpatient intensive care and/or emergency medicine	19 (31.7)
Nonclinical work	2 (3.3)
Research	8 (13.3)
Hospital size, No. of beds	
<300	5 (8.3)
300-499	30 (50.0)
≥500	21 (35.0)
Clinic or outpatient only	4 (6.7)
Clinical role	
Registered nurse	7 (11.7)
Nurse practitioner	3 (5.0)
Attending physician	45 (75.0)
Fellow physician	5 (8.3)
Background in clinical bioethics	
Yes	18 (30.0)
No or do not know	42 (70.0)
Experience in current role, mean (SD), y	17.9 (10.5)
US region	
Pacific coast; 3 states, 12 institutions	37 (61.7)
Midwest and Mountain West; 6 states, 6 institutions	6 (10.0)
Northeast; 4 states, 7 institutions	13 (21.7)
South; 3 states, 4 institutions	4 (6.7)
State deaths per 100 000 residents as of 5/26/2020^c^	
>50	13 (21.7)
10-50	35 (58.3)
<10	12 (20.0)

^a^ One participant did not complete the online survey.

^b^ Clinicians could choose multiple answers.

^c^ The number of deaths was calculated as of May 26, 2020, per Institute for Health Metrics and Evaluation.^26^

Three overlapping and interrelated themes pertaining to resource limitation and clinical care during the COVID-19 pandemic emerged from qualitative analysis, as follows: (1) planning for crisis capacity, (2) adapting to resource limitation, and (3) multiple unprecedented barriers to care delivery. Exemplar quotations are referenced in parenthesis and listed in Table 2, Table 3, and Table 4.

**Table 2.** Exemplar Quotations for Theme 1, Planning for Crisis Capacity

Quotation No.	Participant ID	Participant region	Exemplar quotation
**Developing allocation algorithms**			
1	T	Midwest/Mountain West	The biggest deal in the ethics world in the last 2 months has been preparing in case we need to triage. So, we have a very detailed, elaborate, well thought-out triage policy … that was done at the highest levels of the system.
2	E	Pacific	When there was disagreement … the chair [of the triage team] was like, “let’s all spend some time thinking about it[,]” … and we’ve had the luxury of doing that. I probably have a different opinion than I did last time we talked.
3	P	Pacific	Seeing the varying level of institutional preparedness and support, I’m grateful for what we do have here, because there is a structure, scaffolding, support for frontline clinicians.
4	S	Pacific	Some of the protocols have already been developed from the regional disaster planning[,] … but they’re pretty broad, and how those are going to be actualized are part of the discussion.
5	V	Pacific	They had a real problem with including any sort of age-based criteria in any guidelines because they thought it was using age as a social worth determinant … [but] transplant[ation], and there are many other examples where they’ve use age-based criteria for several decades. It’s just part of survivability. … That’s been 1 of the sticking points in the [state] allocation guidelines.
6	FF	Northeast	From the 50 000-foot view, it sounds great. Yeah, we’re gonna do a renal [kidney] crisis team, working to help people make decisions about CRRT vs not … They were projecting at 1 point that we were going to have a good hundred plus people in need of maybe CRRT machines or ventilators. … Those things, the very granular, like how often should we be doing this, or we can burn out, you know there’s just 3 of us.
7	M	South	Talking to administration, and they just seemed really disengaged with the problem. … We asked multiple times if there was a triage command center or a plan for what would occur if we got to the point where we had to triage resources. They said there was, but they wouldn’t provide it to us. And then I took it upon myself to write my own triage protocol, and my division sent that to them, and we never really got a response.
8	G	Northeast	I was given the ventilator allocation system for our hospital, and immediately it didn’t really feel like, it didn’t feel right. … It seems like a more contemporary approach is to use a multiple principle approach; maximizing life-years as well as lives saved. So, I adapted what I had seen in the [state] guideline.
9	J	Pacific	I think it’s a really good idea. … The triage team, we won’t really know a lot of the characteristics of the people we’re looking at. We’ll know very basics, but there’s things we just don’t want to know, because you’re biased.
10	CC	Pacific	The idea is the practicing clinician gives the triage officer only the information that they need to make this decision. That practically doesn’t make a ton of sense to me. Right? I’m a critical care doctor, and I feel like you have to, you have to get a feel for how sick people are. I think it’s hard to do that without looking in their EHR and laying eyes on them.
11	E	Pacific	The sort of smushy-ness about that is, well, how would we know that we don’t have a nurse, or how would we know that we don’t have a physician? We know we don’t have a bed—that’s pretty easy because there’s not a bed there. But how do you decide if you make that nurse … stay for a second 12 h, or we make that physician stay for the second day? … It’s all is very gray.
12	S	Pacific	So if you come up with a [triage team] policy, it may be well thought out and make a lot of sense … [but] how that is communicated and perceived are also ethical issues, and those also play a role at least in some of the justifications for and against certain policies.
13	E	Pacific	Um, I dunno. Can, am I? Is it okay that I just tell you, I mean, I guess I don’t know. Can I tell you some of the things that we’ve talked about that are, I don’t know how much this, how much, I mean no one’s ever said in these [triage team] meetings that you can’t talk about, I mean well, I guess we kind of have. So, I’ll keep it in big generalities.
14	T	Midwest/Mountain West	There was a lot of very good, very carefully, what’s the word I want, metered information fed down from the incident command structure. … We have never shared it [the triage protocol] with the medical staff as a whole.
15	E	Pacific	I didn’t feel like I should, I could, I should tell anybody … even some of my close friends here who are physicians and nurses here … that I’ve been asked to be on this [triage team]. … I didn’t feel like I should make it known.
**Triage team members**			
16	CC	Pacific	The criteria for choosing a member of the triage team was that they took great critical care nurses and physicians and administrators and an ethicist at each site. And they’re looking for people who are relatively senior and have, or are thought to have, good communication skills.
17	S	Pacific	Not having been in this situation on the triage team before[,] … decisions like this would be very difficult[,] … but I would think it would be similar to some of the ethics committee discussions that we have.
18	L	Northeast	The triage teams were new to me. But … as an ICU doctor you tend to be very comfortable with those types of things. And to just realize that all doctors are going to be having to make triage decisions—not just ICU doctors—that are not used to being put in that situation.
19	E	Pacific	I actually knew nothing about this other than what you read in the *New York Times*. … This is way outside of anything I knew anything about or had any interest in prior to this.
20	CC	Pacific	I do feel the strong sense of duty: that’s what … you do when you’re a physician or health care provider. You step up and respond in these kinds of crises. … I feel like I’m contributing by being willing to do it. It feels rewarding.
21	G	Northeast	It’s like it weighs a lot on you, that this framework could potentially be guiding clinicians to make decisions about who would be able to and who would not be able to receive treatment that they needed. And the thought that someone’s life could be affected by that and that would ripple out to affecting their loved ones’ lives is a lot to deal with.
**Relief that crisis capacity had been averted**			
22	AA	South	I don’t think that’s the purpose of this particular [triage team] group. There might be a group that would want to look at PPE allocation. … It’s a logistics issue not an ethical issue, as I think about it. Whereas, if there aren’t enough ICU beds, it becomes an ethical issue as to who gets it.
23	P	Pacific	I didn’t think it was necessary nor helpful to discuss hypotheticals around crisis standards of care when we are not in crisis standards of care. … There were situations where I encountered questions from the ICU team around the appropriateness of offering CRRT for patients who are extraordinarily ill from COVID-19. I just reminded the team that we are not in crisis standards of care, so the same principles would apply.
24	E	Pacific	We have to do every single thing we can in this contingency phase. … We will not be able to live with ourselves if we haven’t done everything we can to avoid crisis standards of care.
25	CC	Pacific	I’ve actually missed the last couple of [triage team] meetings. … I chose to go for a run. I’m not feeling the same incentive to make a [meeting] call that I didn’t think I was ever going to need to be active on.
26	V	Pacific	There was real urgency when we first started; we wanted to have a tool within 2 or 3 weeks. But then as the surge kept being delayed, and with social distancing, our surge never really materialized to any great extent. … As things went along, we decided we would probably never use this.

Abbreviations: CRRT, continuous renal replacement therapy; EHR, electronic health record; ICU, intensive care unit; PPE, personal protective equipment.

**Table 3.** Exemplar Quotations for Theme 2, Adapting Practices to Limited Resources

Quotation No.	Participant ID	Participant region	Exemplar quotation
**Limited institutional response to resource limitation**			
27	U	Northeast	The main limitation for a long time was really nursing, staffing. … Like everybody, we were worried about ventilator capacity, but that turned out to be sort of, at the end, not the main problem.
28	U	Northeast	Resources were short at various levels all along, some unexpected some more expected. … All of a sudden, we’re out of dialysis catheters, we’re out of central lines, we’re out of A-lines, we’re out of this and out of that. And obviously, I would then kind of have to try to deal with that and see where I could get supplies.
29	M	South	What if we’re okay on ICU beds and vent[ilator]s and staff and all of this stuff, but we’re not okay on dialysis? Do we not have a plan to triage if the rest of the system hasn’t declared that it’s an emergency? It’s still an emergency, right? Like, what happens?
30	M	South	Throughout this whole crisis … the focus has been on vent[ilator]s. Vent[ilator]s and ICU beds, and that’s it. It’s like the whole time, no one has acknowledged that dialysis has been 1 of the most, if not the most, limited resource. It’s just frustrating, the lack of acknowledgment and support.
31	EE	Pacific	We are really close to running out of ventilators. … It felt like we were being hammered and that the rest of the region wasn’t picking up the slack, … just feeling like we were a little bit alone, on an island.
32	Q	Northeast	I emailed all of [my colleagues], and I said “Help! We need x, we need CRRT machines, we need dialysates.” … One of the [hospital’s] attendings had a tweet when we were running out of CRRT. He had a tweet about, “Can anybody give us supplies for CRRT?” So, it got to that. You do anything. You get really desperate.
33	K	Midwest/Mountain West	My partner’s son, he actually borrowed a couple of 3D printers. … He printed some of these face shields, and then they got the formula, or the specifics as to how to make this particular connection to connect to a dialysis machine to generate dialysate. So, he also printed some of those from the 3D printer.
**Redefining standards of care**			
34	W	Northeast	Even the question of having someone die of renal [kidney] failure, that was something that we were not ready to face. So, anything we could do to kind of avoid that, we tried to do.
35	F	Northeast	It was kind of amazing to run out of supplies. A month ago, people said we were going to do acute PD. And I said, “No, we’re not going to do acute PD. PD, it’s not that great for acute patients, sick people in the ICUs. I don’t think we’re going to do PD.” Three days later we were doing acute PD. I mean, that was unbelievable!
36	O	Northeast	Almost like a hackneyed PD catheter, just wait 1 day and boom, start them on PD. … It wasn’t the ideal surgical methodology of starting PD in someone. And they would get complications. There were a few people that couldn’t tolerate it, they’d have a peritoneal leak immediately after or some other problem.
37	AA	South	We never ran out of ventilators, but we definitely had people on travel ventilators, which, would I say that’s the standard of care to manage someone? You can’t tell anything. You just look at the settings pretty much and see the pressures and that’s it. You can’t really tell how they’re interacting with the ventilator and what sort of deleterious lung injury you might be causing.
38	M	South	We were able to get dialysis to everyone who needed it, but I didn’t feel like we were necessarily able to provide enough of a dose of dialysis to make a meaningful contribution to their medical care. We were basically just keeping them hanging on by a thread over the course of the weekend.
39	M	South	We went through the entire list at the beginning of the week and [said], this person has to dialyze these days, this person would probably benefit from a dialysis session, a third group person we could probably just string along and medically manage if we needed to.
40	R	Northeast	No one was not getting dialysis, but there were a lot of people getting minimal dialysis. … Even though people were getting treated, resources were very stretched, and we delayed starting until our hand was forced. … Should you really wait for the potassium to get threateningly high? Probably not.
41	I	Northeast	Two-hour treatments for people with a BUN of 250, you don’t bat an eye at that stuff. It’s like that’s fine, the other person needs it too, or whatever. It’s just because they’re so many. Everybody gets a little bit of bad care.
42	A	Pacific	Severe ARDS and prone and on pressers. They’re all critically ill. There’s no “can we make space?” That wasn’t going to be a possibility. You can’t take what under normal circumstances would receive 1-on-1 nursing care on a ventilator and say, “No, let’s space it out to 2-to-1, or 3-to-1, and also give them a travel vent[ilator].” That’s not a thing and not something we were willing to do.
43	Q	Northeast	We were happy to be able to offer something. That was a positive. As I said, acute PD, we weren’t sure how successful it was going to be, it did allow us to offer something for a period of time.
**Distinguishing clinical care from rationing**			
44	F	Northeast	When you cross that line and say that you’re rationing care, you have done something that is potentially taboo. And that is going to be in the newspapers in a completely different way.
45	AA	South	Under normal times we would’ve been a little more aggressive with saying we’re not going to try to keep doing this because it’s not working, and they’re not getting better. But I think that because of the sensitivity, the concern that people are going to be withholding care and this institution doesn’t want to be seen like that, as a whole we were less likely to have those conversations.
46	Z	Pacific	The treating physicians felt terribly conflicted about making resource decisions. But it was rationing, let’s call it what it was! It’s like the Scribner days, “who shall live and who shall die” without dialysis. So, the chief medical officer made rounds with them and he made a call. … You have 3 patients and you can dialyze 1. And [he] made basically judgment calls that were best medical judgment. … It was pretty arbitrary.
47	X	Northeast	Everybody got done that we wanted dialysis on in the end. Yeah, I guess we were lucky in that way. I mean … there were some K’s of 7s that got us very worried.
48	F	Northeast	If you cross that line, it’s called rationing. … If I make a choice, and say, “That guy is clearly not going to make it,” I’m practicing medicine. But if I said, “Okay, here are the criteria that I’m going to apply, the criteria are made by a group of individuals, which includes an attorney, an intensivist, and a nephrologist.” Then I say, “Okay, I’m not going to dialyze people who are chronic hemodialysis patients or dialyze 80 year olds. Oh, hemodialysis patients, they’re Black, they’re minorities, those are people of color, I’m not dialyzing them.” What do you do then? That’s the line. … We all think practicing medicine includes taking into account comorbidity.
49	M	South	They felt that this patient had the greatest likelihood of benefitting from most aggressive therapy. … I think there was probably like 5 or 6 patients in the ICU … and then you had this 35-year-old with no comorbidities. That’s who the ICU dialyzed, and I couldn’t really disagree.
50	V	Pacific	I’m not sure how other specialties and other areas of medicine triage or allocate resources, but I feel like most intensivists probably do it on a daily basis. … I feel like I kind of do this at a microlevel as part of my normal practice. And it’s not because I would say resources are scarce, it’s because it’s what’s going to be a meaningful benefit to the patient, so it’s in the idea of futile or inappropriate care. For me, it feels pretty comfortable.
51	X	Northeast	I don’t like to use the word rationing, but when you’re, I mean, in a normal situation there’s like sort of expected dialysis. Like, if you’re an ESRD patient, you generally would get it 3 times a week. … It’s just very different than when you’re resource poor.
52	O	Northeast	When you have 3 or 4 people, all in their 60s, all diabetic with ESRD, all on BiPAP and hypoxic, all with potassiums of 5.4, you’re kind of reaching a point where you don’t have much clinical tools to guide you about choosing between those people, who needs the dialysis first. … It was getting to the point where these decisions were becoming arbitrary and not based on any real clinical reasoning.
53	O	Northeast	We were stuck making decisions between a bunch of people who were just all overloaded. We’d kind of make a judgement call based on the degree of hypoxia and, I hate to say it, but just their age and comorbidities.
54	X	Northeast	Not that we are rationing dialysis, but we did have to decide in the day-to-day, like who was going to die without it. And so I think that, it is, I mean I guess, age sort of trumps most things. But that’s also hard.
**Moral distress**			
55	M	South	I was hearing about how limited the resources were in the hospital. I was horrified, and I was terrified about having to make these decisions. I mean, I couldn’t sleep at night.
56	N	Pacific	Like, I wasn’t doing a great job as a doctor. Because you want to provide the best care possible, right? To each and every patient. It’s not always possible, I guess.
57	C	Pacific	I don’t think I can confidently say I’ve given all of my patients the care that they need right now or in the last 2 months. I think I have, but would I be shocked to find out in a month or 2 that a patient had an iatrogenic complication from a medication or something that I probably would’ve picked up if they didn’t have that May or April clinic visit canceled?
58	O	Northeast	If you keep thinking about what you are doing, that, it can really mess you up. I won’t lie, I cried a couple of times walking home from work. … I was starting to worry that I was going to be making a decision between people that clinically needed dialysis equally and just arbitrarily choosing who’s going to be the one who will get it that day and who’s going to be pushed. And then if something happened to the one that was pushed to the next day, it almost feels like, who are you? Are you a doctor or are you an angel of death who’s making arbitrary decision on who lives and dies?
59	H	Pacific	At that time, the guidance wasn’t there, so it felt a little bit like I was shirking duty by not going in the room. … Are we really able to provide the same clinical care without actually physically seeing the patient? … It’s reassuring that the guidance is don’t do it.
60	DD	Pacific	We feel very strongly that we know what’s right for the patient … and when you take that decision-making away [about how to use limited COVID-19 tests] … who is responsible for wrong here? Who’s really responsible? Am I responsible? I’m going to feel responsible. … I’m taking on all that responsibility because that’s what I do. That’s my job. All that’s my job. But you as infection prevention, you can stand there and say, “Well, we were following the guidelines.”

Abbreviations: 3D, 3-dimensional; A-line, arterial line; ARDS, acute respiratory distress syndrome; BiPAP, Bilevel positive airway pressure; BUN, blood urea nitrogen; COVID-19, coronavirus disease 2019; CRRT, continuous renal replacement therapy; ESRD, end-stage renal disease; ICU, intensive care unit; K, potassium; PD, peritoneal dialysis.

**Table 4.** Exemplar Quotations for Theme 3, Multiple Unprecedented Barriers to Care Delivery

Quotation No.	Participant ID	Participant region	Exemplar quotation
**Contact limitation**			
61	CC	Pacific	It takes a while to get in there, and if someone starts pulling on their ET tube, there’s, pre-COVID[-19], there’s often a nurse in the room. … It is much better for patients to have a RASS of 0 or 1, and our patients all have a RASS −4. That’s all we have to do under these circumstances. … The alternative is to not have them in isolation, which is not a feasible alternative.
62	Y	Pacific	I’ve been talking to them on the phone, outside the room, and I’m like, “Here I am!” But definitely, you don’t feel the connection with your patient like it used to be. … The patient’s so tearful, so anxious about going back home. And I think it would’ve made a big difference, had I been at the patient’s bedside.
63	K	Midwest/Mountain West	There’s other patients in the hospital who don’t have COVID[-19], or who are not under investigation, and there’s no reason why you shouldn’t be there seeing those patients. If that’s the case, if you don’t want to have contact with patients because of this … then you should become a pathologist. But, that’s my opinion.
64	D	Pacific	People had been shamed for wearing masks a few weeks ago, and then I wondered if it was some kind of, “I’m not going to use PPE,” like, it was just for weak people. I’m not sure. But I was really shocked. … They were all sitting around talking, and I walked by with a mask, and it almost seemed like they kind of looked at me funny.
65	EE	Pacific	I call her [a patient’s wife] and say, “Unfortunately, he’s on 100%, I would have to put him on the ventilator.” And she says, “Absolutely not, he’s not in distress.” So, she can’t see him. … I said to her, “But I’m an expert! I can tell you he needs to be intubated.” And she says, “No, he’s not in distress.” … Normally having families there, they see how often you’re in that room trying to take care. I think I was in that room for hours and hours that day, and so they build that trust.
66	DD	Pacific	[I remember] looking at the ICU physician and being like, “Have you talked to the son this week?” And she’s like, “Oh my God, no. … Did you talk to the son?” I’m like, “Oh my God, no.” And realizing that none of us had called the family because it’s just not in your workflow. You’re so used to the family being there.
**Rapid pace of change and uncertainty**			
67	L	Northeast	I was getting multiple emails, multiple times a day regarding best practices and various new articles that were coming out. … This is what we’re going to be doing for high-flow nasal canula, and noninvasive positive pressure, and now we’re going to reuse our N-95s. So just lots of things rapidly evolving.
68	C	Pacific	There’s just constant stuff, right? There’s news from medical journals, news from reports from other cities and what their experiences have been. There’s projections upon projections upon projections. And I think all of that amounts to this incredible torrent.
69	L	Northeast	[The intensivists] were happy with any kind of unproven therapy, even if there were risks. I think they were desperate to also give families good news, even though things looked grim. There was just a lot of desperation to keep patients alive, probably because they’d been so traumatized by patients unexpectedly dying.
70	T	Midwest/Mountain West	The palliative care team, we thought we’d just be swamped. … We really got almost no consults on the COVID[-19] patients. … We’ll get the consults the moment the COVID[-19] test comes back negative. Like, “Ding! Negative. Palliative consult.” … Perhaps the hospitalists … are uncomfortable consulting palliative care on a disease where you really don’t know the outcomes yet and are concerned that they may be sending a very negative message. “Oh gee, it’s COVID-19. We need palliative care.”
71	EE	Pacific	It’s a really weird disease, and I think that’s the hard part, being able to prognosticate. … I think that’s getting harder for us, to say anything with certainty. Unless someone’s coding, and even then, it behaves differently. … I think as a group, we are a lot slower to have some of those big [goals of care] discussions.
**Discussions with families about disrupted care**			
72	B	Pacific	Most families have been actually very understanding. This is a crisis, and we’re in a pandemic, and we’re all doing things we wouldn’t normally do.
73	W	Northeast	We were pretty honest about how resources were limited and how we were doing with this COVID[-19] surge. And I think we talked about how, the usual, you know, ability to provide aggressive dialysis was not the case with COVID[-19]. … There was a lot of understanding, sometimes to my surprise. … I would think people would be more upset when hearing something like that.
74	AA	South	I was actually expecting having to do some deescalation and some heated discussions. … [I] explained to the families also that it wasn’t just to protect the patient’s comfort and to not do something for them that wouldn’t be beneficial, but also for the medical providers who would have to be in the high risk situation like a code. … The families were quite receptive to that and felt that they didn’t want to be putting health care workers at risk either.
75	M	South	I didn’t bring up resource limitation on the phone; that’s not appropriate with a patient’s family, but I think they sensed that was going on. Somehow they picked up on that, and they got very upset with my suggestion that maybe we forego dialysis knowing that his mortality was very high. Like, it just sucked, because in general I feel like I do these conversations pretty well, and partially, looking back on it, I think in part it was maybe my own anxiety around the situation, the conversation. I remember the lady saying I sounded “rushed[,]” … sounded “detached.” … I remember being very ashamed about the conversation.
76	O	Northeast	I kind of had a deal with telling him, “Listen, you’re too stable for dialysis right now,” even though if not in a pandemic he would’ve gotten dialyzed 2 days ago and today, but that we’re pushing him again. He was just extremely frustrated. He said, “This is crazy, what is this?” He was like mad at me personally. All I could do was try to explain our perspective here, that we’re overwhelmed. This is the situation we’re in. This is an international, this is a pandemic. … I don’t think it means much to someone who’s supposed to get dialysis and they’re short of breath, being denied the treatment they need.
77	G	Northeast	There was so much that was unknown about the trajectory, and also, … we didn’t know, are we going to run out of dialysis fluid at some point? … We left it as, their loved one was very sick, and it needed to be a day-by-day conversation with the ICU team. Just to make sure that we weren’t causing more harm or difficulties than benefit with them. I think that’s more how we left it.

Abbreviations: COVID-19, coronavirus disease 2019; ET tube, endotracheal tube; ICU, intensive care unit; PPE, personal protective equipment; RASS, Richmond Agitation-Sedation Scale.

### Planning for Crisis Capacity

Institutional leaders who participated in planning for crisis capacity described the challenges of adapting and operationalizing existing guidelines as well as the substantial moral weight of the task. They were relieved when it became clear that these processes would likely not be needed at their institutions (Table 2).

#### Developing Allocation Algorithms

Clinicians who were involved in institutional planning described strong institutional support for their work to develop protocols to guide care should their region reach crisis capacity (quotation 1). They believed that establishing protocols in advance would allow for a more carefully considered approach (quotation 2) and would be reassuring to staff (quotation 3).

Their work involved developing actionable triage algorithms based on existing frameworks (quotation 4). Group deliberation focused on both big picture ethical questions (quotation 5) as well as more granular operational details (quotation 6). Several clinicians with whom we spoke were not formally involved in institutional planning efforts but had offered input and/or developed their own protocols when they identified gaps in planning or disagreed with institutional policies (quotations 7 and 8).

To support fairness and avoid bias, the plan was for triage team members to receive very limited information about individual patients when making selection decisions (quotation 9). Some clinicians involved in planning were skeptical about the feasibility of making triage decisions in the absence of detailed clinical information (quotation 10). Others expressed uncertainty about whether and how triage protocols would address more ambiguous or dynamic types of resource limitation (eg, ICU bed shortages vs staff or supply shortages) (quotation 11). Clinicians could also be mindful of how their work might be viewed by other clinicians and the public (quotation 12) and were wary of sharing details about committee deliberations (quotation 13), plans (quotation 14), or team membership (quotation 15) with their colleagues and/or during the research interview.

#### Triage Team Members

Clinicians who had been appointed to triage teams were usually respected leaders in intensive care, palliative care, or bioethics who were recognized for their ability to collaborate and communicate (quotation 16). Some of the intensivists and ethicists with whom we spoke saw the work of the triage team as an extension of their usual work (quotations 17 and 18), but many clinicians saw this experience as entirely new (quotation 19). Clinicians described being motivated to participate in the triage team out of a sense of duty and desire to contribute (quotation 20) but were also cognizant of the moral weight and emotional burden of the task before them (quotation 21).

#### Relief that Crisis Capacity Had Been Averted

Clinicians involved in triage planning understood the processes they were developing to be intended exclusively for crisis settings (quotations 22 and 23) and saw the importance of optimizing resources under contingency capacity to avoid having to resort to crisis standards of care (quotation 24). While several clinicians described a period of intense planning early in the pandemic, by the time of our interviews, many were relieved to report that crisis standards of care were unlikely to be invoked at their institutions, and some had paused or disengaged from triage planning (quotations 25 and 26).

### Adapting to Resource Limitation

Clinicians working during the pandemic were forced to grapple with multiple expected and unexpected forms of resource limitation that did not rise to the level of triggering crisis capacity. Nevertheless, these limitations could compromise care, require that they make difficult allocation decisions, and engender moral distress (Table 3).

#### Limited Institutional Response to Resource Limitation

Although none of the clinicians with whom we spoke reported a shift to crisis capacity at their institutions, they nevertheless described being faced with a range of expected and unexpected forms of resource limitation (eg, dialysis machines, staff, routine supplies) (quotation 27) that could arise in a haphazard manner with little warning (quotation 28). Some expressed frustration that resource shortages they were seeing in practice were not acknowledged as such by hospital or regional leadership (quotations 29 and 30) or felt unsupported by colleagues at neighboring medical centers (quotation 31). When not available from their institutions, some clinicians resorted to obtaining health care equipment through personal contacts or even fabricated it themselves (quotations 32 and 33).

#### Redefining Standards of Care

Clinicians were strongly motivated to avoid situations in which they would have to categorically deny needed treatment to any patient (quotation 34) and went to great lengths to develop alternative treatment options (quotation 35). This might involve using unorthodox therapies or nontraditional approaches to care delivery that could be suboptimal or potentially harmful (quotations 36 and 37). For example, some nephrologists described triaging patients for hemodialysis based on immediate need (quotations 38 and 39), delaying dialysis until there was an emergent indication (quotation 40), and/or prescribing shorter treatment times. As 1 clinician explained, “everyone gets a little bit of bad care” (quotation 41). Rarely, clinicians were able to draw a clear line between acceptable and unacceptable care (quotation 42), but most focused on doing the best they could under the circumstances (quotation 43).

#### Distinguishing Clinical Care From Rationing

The notion of rationing generally had negative connotations (quotation 44), and some clinicians even described erring on the side of providing more intensive interventions to avoid the appearance of rationing (quotation 45). With rare exceptions (quotation 46), clinicians did not explicitly speak of having had to ration health care resources (quotation 47). Nonetheless, some clinicians did describe situations in which they had to make difficult choices about which patients would and would not receive life-saving therapies, typically on the basis of age and/or comorbidity. This was often seen as part of the spectrum of normal clinical decision-making (quotations 48 and 49) and within their scope of practice (quotation 50). Some clinicians did describe struggling to understand what constituted acceptable practice vs rationing (quotations 51 and 52) and expressed misgivings about the approach to selecting patients for life-saving treatments during the pandemic (quotations 53 and 54).

#### Moral Distress

Many clinicians were fearful of having to ration resources (quotation 55). Even in lower acuity and outpatient settings, some struggled with whether it was acceptable to provide suboptimal care (quotation 56) and worried about the potential harms of disrupted care practices (quotation 57). A sense of responsibility for poor outcomes could take a substantial emotional toll (quotation 58). Some clinicians felt that explicit guidelines would be helpful in limiting this moral distress (quotation 59), while others felt personally responsible for poor outcomes regardless of whether they were adhering to institutional recommendations or requirements (quotation 60).

### Multiple Unprecedented Barriers to Care Delivery

Clinicians described multiple barriers to care delivery during the pandemic. These challenges compounded and were difficult to disentangle from the effects of resource limitation (Table 4).

#### Contact Limitation

Policies and practices were modified to limit physical interaction between staff, patients, and family members with the dual goals of reducing viral spread and conserving personal protective equipment. While necessary, policies to limit contact with patients were seen as being detrimental to care and to the experiences of both patients and clinicians (quotations 61 and 62). Decisions about something as routine as whether to perform a physical examination could expose tensions around the conflicting goals of conserving scarce resources, protecting oneself, and caring for patients (quotations 63 and 64). Visitor restrictions could complicate and disrupt the process of engaging family members in decision-making (quotations 65 and 66).

#### Rapid Pace of Change and Uncertainty

The rapid pace of change and limited scientific understanding of COVID-19 (quotations 67 and 68) led to substantial uncertainty in day-to-day practice. A sense of desperation and a desire to do everything possible to save lives might lead to more aggressive treatment practices and/or greater willingness to try unproven therapies (quotation 69), reluctance to engage palliative care specialists (quotation 70), and delays in end-of-life decision-making (quotation 71).

#### Discussions With Families About Disrupted Care

Many clinicians commented that families and patients were often quite understanding of care disruption (quotation 72), and that they were surprised at how accepting some families could be when care was compromised by the need to conserve resources and/or protect clinicians (quotations 73 and 74). However, some clinicians did describe contentious interactions with family members (quotation 75) and patients (quotation 76) who felt that care was being inappropriately withheld. Other clinicians described deliberately avoiding mention of resource limitation when talking with families (quotation 77).

## Discussion

Our thematic analysis of interviews with US clinicians who were directly involved in patient care and/or strategic planning during the COVID-19 pandemic highlights the real-world complexity of adaptation to resource limitation. Clinicians described patterns of institutional planning that mirrored the IOM’s phased approach, which assumes a common understanding about what constitutes usual standards of care with a plan for a coordinated regional response when these become untenable. However, consistent with prior anecdotal reports,^27,28,29,30^ the clinicians we interviewed described how, even in the absence of formal declarations of crisis capacity, a variety of expected and unexpected forms of resource limitations severely compromised care and required that they make difficult allocation decisions at the bedside.

Clinicians and clinical teams went to great lengths to develop alternative treatment options and to stretch existing resources to provide at least some care to all in need and avoid having to categorically deny treatment to any patient. However, this approach could mean providing care that fell far short of the IOM standard of functional equivalence to usual care. When substituting lower-quality or delayed treatments, clinicians and clinical teams were left to grapple with what constituted an acceptable standard of care, which could be a source of self-doubt and moral distress. While most clinicians did not feel that they had been in the position of having to ration scarce resources, some nevertheless described practices, such as selection by age or comorbidity, that may be subject to implicit biases^31^ and may not be supported by societal priorities for fairness in resource allocation.^32,33,34,35^

Existing frameworks guiding institutional approaches to resource allocation in crisis settings^4,5,6,7^ represent an important step toward promoting fairness through transparent allocation processes.^36^ However, our results suggest that a narrow focus on crisis capacity may fail to address the full spectrum and complexity of challenges to providing high-quality care encountered by frontline clinicians during the COVID-19 pandemic (Figure). Insights gained from our study suggest several strategies to better support clinicians and guide clinical care as the pandemic evolves. First, regional authorities and institutions could prepare guidelines, protocols, and defined standards of care in advance to address the myriad types of resource limitation and challenges to providing high-quality care that arise long before declaration of crisis capacity. Second, in addition to their narrow role in allocating scarce resources under crisis capacity, triage team members could collaborate and/or consult with frontline clinicians to address challenges related to more nuanced forms of resource limitation.^37,38^ Finally, as the community moves beyond the current crisis and plans for the future, training in bioethics may help to support clinical teams in navigating the value conflicts that can arise when resources are limited in both pandemic and usual care settings.^39,40^

**Figure.**
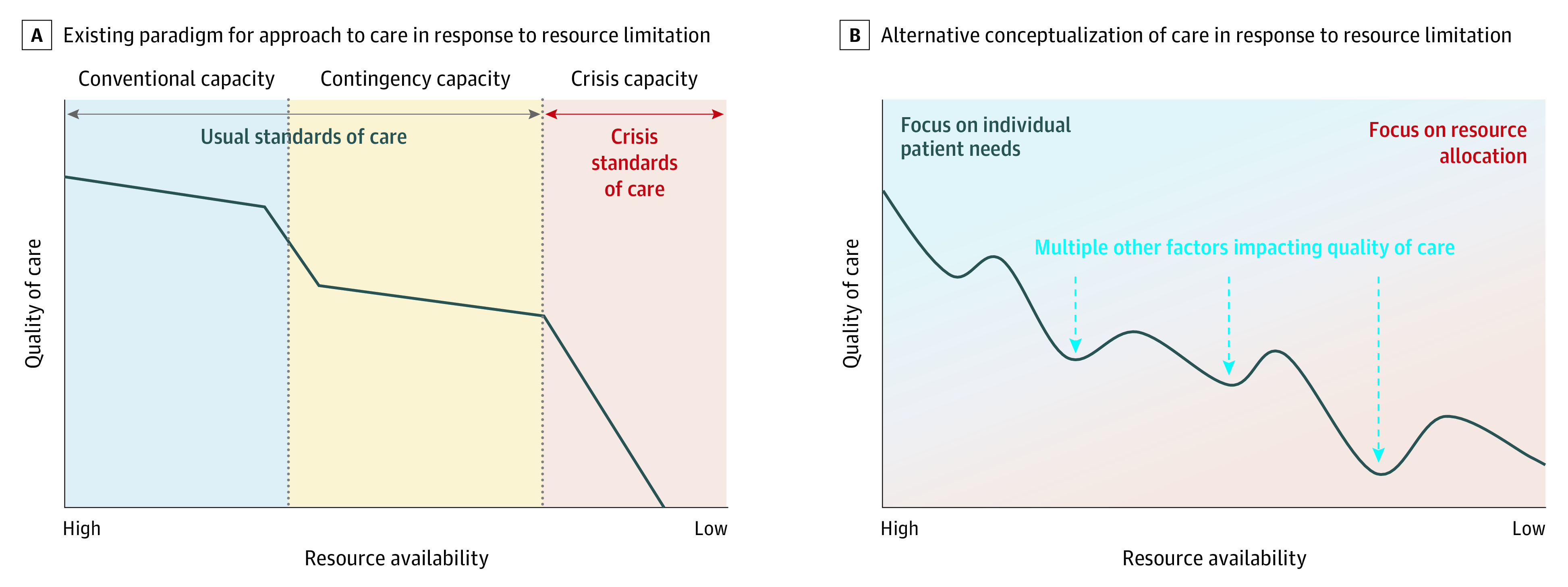
Conceptual Frameworks Describing Approaches to Health Care Resource Limitation and Impact on Quality of Care A, Existing paradigmatic approach to health care resource limitation based on the Institute of Medicine’s *Crisis Standards of Care: A Systems Framework for Catastrophic Disaster Response*.^4^ B, Description of response to resource limitation based on our analysis of clinician experience during the coronavirus disease 2019 pandemic. Multiple other factors that compounded the association of resource limitation with quality of care during the pandemic included the need to limit contact between clinicians and restrict visitors, the rapid pace of change, and the lack of scientific evidence.

### Limitations

This study has limitations. Our results may not capture the experiences and perspectives of clinicians practicing in other parts of the world, in regions of the United States not represented in our study, or in specialties not represented by our participants (eg, pediatrics).^41^ We also did not collect information about the COVID-19 caseload at each participant’s institution at the time of the interview, which may have shaped participants’ experiences and perspectives. Because resource allocation can be a sensitive topic, participants may have felt limited in discussing some aspects of their experiences. Furthermore, the dynamic nature of the pandemic makes it likely that new challenges not identified in our study will arise over time and practices may evolve in response to early experience.

## Conclusions

In this qualitative study, many clinicians described institutional planning for crisis capacity, but this did not always address real-world challenges to providing care when resources were limited. Expanding the scope of institutional planning beyond crisis capacity may be helpful in supporting clinicians and addressing moral distress, promoting equity, and optimizing care as the pandemic evolves.

## Notes

Supplement. **eTable 1.** Consolidated Criteria for Reporting Qualitative Research Guidelines (COREQ) Checklist **eTable 2. **Sample Semistructured Interview Guide

## References

[1] Holshue ML, DeBolt C, Lindquist S, ; Washington State 2019-nCoV Case Investigation Team First case of 2019 novel coronavirus in the United States. N Engl J Med. 2020;382(10):929-936. doi:10.1056/NEJMoa2001191 32004427 PMC7092802

[2] Rosenbaum L Facing COVID-19 in Italy—ethics, logistics, and therapeutics on the epidemic’s front line. N Engl J Med. 2020;382(20):1873-1875. doi:10.1056/NEJMp2005492 32187459

[3] White DB, Lo B A framework for rationing ventilators and critical care beds during the COVID-19 pandemic. JAMA. 2020;323(18):1773-1774. doi:10.1001/jama.2020.5046 32219367

[4] Committee on Guidance for Establishing Crisis Standards of Care for Use in Disaster Situations, Institute of Medicine Crisis Standards of Care: A Systems Framework for Catastrophic Disaster Response. National Academies Press; 2012. 24830057

[5] Christian MD, Devereaux AV, Dichter JR, Rubinson L, Kissoon N; Task Force for Mass Critical Care; Task Force for Mass Critical Care Introduction and executive summary: care of the critically ill and injured during pandemics and disasters: CHEST consensus statement. Chest. 2014;146(4)(suppl):8S-34S. doi:10.1378/chest.14-0732 25144202 PMC7094437

[6] World Health Organization Pandemic influenza preparedness. Accessed June 26, 2020. https://apps.who.int/gb/pip/

[7] Pandemic Influenza Ethics Initiative Work Group of the Veterans Health Administration’s National Center for Ethics in Health Care Meeting the challenge of pandemic influenza: ethical guidance for leaders and healthcare professionals in the Veterans Health Administration. July 2010.

[8] White DB, Katz MH, Luce JM, Lo B Who should receive life support during a public health emergency? using ethical principles to improve allocation decisions. Ann Intern Med. 2009;150(2):132-138. doi:10.7326/0003-4819-150-2-200901200-00011 19153413 PMC2629638

[9] Bell JA, Hyland S, DePellegrin T, Upshur RE, Bernstein M, Martin DK SARS and hospital priority setting: a qualitative case study and evaluation. BMC Health Serv Res. 2004;4(1):36. doi:10.1186/1472-6963-4-36 15606924 PMC544195

[10] Singer PA, Benatar SR, Bernstein M, Ethics and SARS: lessons from Toronto. BMJ. 2003;327(7427):1342-1344. doi:10.1136/bmj.327.7427.1342 14656848 PMC286332

[11] Daugherty Biddison EL, Gwon H, Schoch-Spana M, The community speaks: understanding ethical values in allocation of scarce lifesaving resources during disasters. Ann Am Thorac Soc. 2014;11(5):777-783. doi:10.1513/AnnalsATS.201310-379OC 24762135

[12] Cheung W, Myburgh J, McGuinness S, ; Influenza Pandemic ICU Triage 3 study investigators; Australian and New Zealand Intensive Care Society Clinical Trials Group A cross-sectional survey of Australian and New Zealand public opinion on methods to triage intensive care patients in an influenza pandemic. Crit Care Resusc. 2017;19(3):254-265. 28866976

[13] Maunder R, Hunter J, Vincent L, The immediate psychological and occupational impact of the 2003 SARS outbreak in a teaching hospital. CMAJ. 2003;168(10):1245-1251. 12743065 PMC154178

[14] Nickell LA, Crighton EJ, Tracy CS, Psychosocial effects of SARS on hospital staff: survey of a large tertiary care institution. CMAJ. 2004;170(5):793-798. doi:10.1503/cmaj.1031077 14993174 PMC343853

[15] Liu Q, Luo D, Haase JE, The experiences of health-care providers during the COVID-19 crisis in China: a qualitative study. Lancet Glob Health. 2020;8(6):e790-e798. doi:10.1016/S2214-109X(20)30204-7 32573443 PMC7190296

[16] Dreifuss BA I’m a health care worker. You need to know how close we are to breaking. *New York Times* Published June 26, 2020. Accessed October 9, 2020. https://www.nytimes.com/2020/06/26/opinion/coronavirus-arizona-hospitals.html?searchResultPosition=4

[17] Emanuel EJ, Phillips J, Persad G How the coronavirus may force doctors to decide who can live and who dies. *New York Times* Published March 12, 2020. Accessed October 9, 2020. https://www.nytimes.com/2020/03/12/opinion/coronavirus-hospital-shortage.html

[18] Shanafelt T, Ripp J, Trockel M Understanding and addressing sources of anxiety among health care professionals during the COVID-19 pandemic. JAMA. 2020;323(21):2133-2134. doi:10.1001/jama.2020.5893 32259193

[19] Hasan Z, Narasimhan M Preparing for the COVID-19 pandemic: our experience in New York. Chest. 2020;157(6):1420-1422. doi:10.1016/j.chest.2020.03.027 32222587 PMC7152895

[20] Sharma A, Maxwell CR, Farmer J, Greene-Chandos D, LaFaver K, Benameur K Initial experiences of US neurologists in practice during the COVID-19 pandemic via survey. Neurology. 2020;95(5):215-220. doi:10.1212/WNL.0000000000009844 32439820

[21] Sharma M, Creutzfeldt CJ, Lewis A, Healthcare professionals’ perceptions of critical care resource availability and factors associated with mental well-being during COVID-19: Results from a US survey. Clin Infect Dis. 2020;2(Sep):ciaa1311. doi:10.1093/cid/ciaa1311 32877508 PMC7499503

[22] Tong A, Sainsbury P, Craig J Consolidated criteria for reporting qualitative research (COREQ): a 32-item checklist for interviews and focus groups. Int J Qual Health Care. 2007;19(6):349-357. doi:10.1093/intqhc/mzm042 17872937

[23] Braun V, Clarke V Using thematic analysis in psychology. Qualitative Research in Psychology. 2006;3(2):77-101. doi:10.1191/1478088706qp063oa

[24] Birks M, Mills J. Grounded Theory: A Practical Guide. 2nd ed. Sage Publications Inc; 2015.

[25] Giacomini MK, Cook DJ; Evidence-Based Medicine Working Group Users’ guides to the medical literature: XXIII. qualitative research in health care A. are the results of the study valid? JAMA. 2000;284(3):357-362. doi:10.1001/jama.284.3.357 10891968

[26] Institute for Health Metrics and Evaluation COVID-19 projections. Accessed July 30, 2020. https://covid19.healthdata.org/global?view=total-deaths&tab=trend

[27] Goldfarb DS, Benstein JA, Zhdanova O, Impending shortages of kidney replacement therapy for COVID-19 patients. Clin J Am Soc Nephrol. 2020;15(6):880-882. doi:10.2215/CJN.05180420 32345750 PMC7274293

[28] Stanworth SJ, New HV, Apelseth TO, Effects of the COVID-19 pandemic on supply and use of blood for transfusion. Lancet Haematol. 2020;7(10):e756-e764. doi:10.1016/S2352-3026(20)30186-1 32628911 PMC7333996

[29] Abbasi J “Abandoned” nursing homes continue to face critical supply and staff shortages as COVID-19 toll has mounted. JAMA. 2020;324(2):123-125. doi:10.1001/jama.2020.10419 32525535

[30] Powell T, Chuang E COVID in NYC: what we could do better. Am J Bioeth. 2020;20(7):62-66. doi:10.1080/15265161.2020.1764146 32464081

[31] Chapman EN, Kaatz A, Carnes M Physicians and implicit bias: how doctors may unwittingly perpetuate health care disparities. J Gen Intern Med. 2013;28(11):1504-1510. doi:10.1007/s11606-013-2441-1 23576243 PMC3797360

[32] Nord E, Richardson J, Street A, Kuhse H, Singer P Maximizing health benefits vs egalitarianism: an Australian survey of health issues. Soc Sci Med. 1995;41(10):1429-1437. doi:10.1016/0277-9536(95)00121-M 8560311

[33] Tong A, Howard K, Jan S, Community preferences for the allocation of solid organs for transplantation: a systematic review. Transplantation. 2010;89(7):796-805. doi:10.1097/TP.0b013e3181cf1ee1 20090570

[34] Ubel PA, Loewenstein G Distributing scarce livers: the moral reasoning of the general public. Soc Sci Med. 1996;42(7):1049-1055. doi:10.1016/0277-9536(95)00216-2 8730910

[35] Farrell TW, Francis L, Brown T, Rationing limited healthcare resources in the COVID-19 era and beyond: ethical considerations regarding older adults. J Am Geriatr Soc. 2020;68(6):1143-1149. doi:10.1111/jgs.16539 32374466 PMC7267288

[36] Daniels N, Sabin JE Setting Limits Fairly, Learning to Share Resources for Health. 2nd ed. Oxford University Press; 2008.

[37] Alexander GC, Werner RM, Ubel PA The costs of denying scarcity. Arch Intern Med. 2004;164(6):593-596. doi:10.1001/archinte.164.6.593 15037486

[38] Berlinger N, Wynia M, Powell T, Ethical framework for health care institutions and guidelines for institutional ethics services responding to the coronavirus pandemic: managing uncertainty, safeguarding communities, guiding practice. Published March 16, 2020 Accessed October 9, 2020. https://www.thehastingscenter.org/ethicalframeworkcovid19/

[39] Carrese JA, Sugarman J The inescapable relevance of bioethics for the practicing clinician. Chest. 2006;130(6):1864-1872. doi:10.1378/chest.130.6.1864 17167010

[40] Dudzinski DM, Hoisington BY, Brown CE Ethics lessons from Seattle’s early experience with COVID-19. Am J Bioeth. 2020;20(7):67-74. doi:10.1080/15265161.2020.1764137 32552455

[41] Jansen M, Irving H, Gillam L, Ethical considerations for paediatrics during the COVID-19 pandemic: a discussion paper from the Australian Paediatric Clinical Ethics Collaboration. J Paediatr Child Health. 2020;56(6):847-851. doi:10.1111/jpc.14946 32471008 PMC7300784

