# Supplementary material for: US Clinicians’ Experiences and Perspectives on Resource Limitation and Patient Care During the COVID-19 Pandemic
Source: JAMA Netw Open. 2020 Nov 6;3(11):e2027315. doi: 10.1001/jamanetworkopen.2020.27315 (PMC7648254; doi:10.1001/jamanetworkopen.2020.27315)
Supplement: Supplement. — eTable 1. Consolidated Criteria for Reporting Qualitative Research Guidelines (COREQ) Checklist eTable 2. Sample Semistructured Interview Guide [file jamanetwopen-e2027315-s001.pdf]

# Supplemental Online Content

Butler CR, Wong SPY, Wightman AG, O'Hare AM. US clinicians' experiences and perspectives on resource limitation and patient care during the COVID-19 pandemic. *JAMA Netw Open*. 2020;3(11):e2027315. doi:10.1001/jamanetworkopen.2020.27315

**eTable 1.** Consolidated Criteria for Reporting Qualitative Research Guidelines (COREQ) Checklist
**eTable 2.** Sample Semistructured Interview Guide

This supplemental material has been provided by the authors to give readers additional information about their work.

© 2020 Butler CR et al. *JAMA Network Open.*

**eTable 1.** Consolidated Criteria for Reporting Qualitative Research Guidelines (COREQ) Checklist

| Item | Guide questions/description | Location in manuscript |
|---|---|---|
| **Domain 1: Research team and reflexivity** | | |
| *Personal Characteristics* | | |
| 1. Interviewer/facilitator | Which author/s conducted the interview or focus group? | Methods, Paragraph 2 |
| 2. Credentials | What were the researcher's credentials? E.g. PhD, MD | Author list |
| 3. Occupation | What was their occupation at the time of the study? | Methods, Paragraph 2 |
| 4. Gender | Was the researcher male or female? | Methods, Paragraph 2 |
| 5. Experience and training | What experience or training did the researcher have? | Methods, Paragraph 2 |
| *Relationship with participants* | | |
| 6. Relationship established | Was a relationship established prior to study commencement? | Methods, paragraph 1 |
| 7. Participant knowledge of the interviewer | What did the participants know about the researcher? e.g. personal goals, reasons for doing the research | The intention of the research (to better understand clinician experience during the pandemic) was included in written information materials. |
| 8. Interviewer characteristics | What characteristics were reported about the interviewer/facilitator? e.g. Bias, assumptions, reasons and interests in the research topic | Methods, paragraph 2 |
| **Domain 2: Study design** | | |
| *Theoretical framework* | | |
| 9. Methodological orientation and Theory | What methodological orientation was stated to underpin the study? e.g. grounded theory, discourse analysis, ethnography, phenomenology, content analysis | Methods, paragraph 3. |
| *Participant selection* | | |
| 10. Sampling | How were participants selected? e.g. purposive, convenience, consecutive, snowball | Methods, paragraph 1 |
| 11. Method of approach | How were participants approached? e.g. face-to-face, telephone, mail, email | Methods, paragraph 1 |
| 12. Sample size | How many participants were in the study? | Results, paragraph 1 |
| 13. Non-participation | How many people refused to participate or dropped out? Reasons? | Results, paragraph 1 |
| *Setting* | | |
| 14. Setting of data collection | Where was the data collected? e.g. home, clinic, workplace | Methods, paragraph 2 |
| 15. Presence of non-participants | Was anyone else present besides the participants and researchers? | No. |
| 16. Description of sample | What are the important characteristics of the sample? e.g. demographic data, date | Results, paragraph 1 |

© 2020 Butler CR et al. *JAMA Network Open.*

| | | |
|---|---|---|
| *Data collection* | | |
| 17. Interview guide | Were questions, prompts, guides provided by the authors? Was it pilot tested? | Appendix, Table 1 |
| 18. Repeat interviews | Were repeat interviews carried out? If yes, how many? | Methods, paragraph 2 |
| 19. Audio/visual recording | Did the research use audio or visual recording to collect the data? | Methods, paragraph 1 |
| 20. Field notes | Were field notes made during and/or after the interview or focus group? | Yes. |
| 21. Duration | What was the duration of the interviews or focus group? | Methods, paragraph 1 |
| 22. Data saturation | Was data saturation discussed? | Methods, paragraph 3 |
| 23. Transcripts returned | Were transcripts returned to participants for comment and/or correction? | Methods, paragraph 2 |
| **Domain 3: Analysis and findings** | | |
| *Data analysis* | | |
| 24. Number of data coders | How many data coders coded the data? | Methods, paragraph 3 |
| 25. Description of the coding tree | Did authors provide a description of the coding tree? | Method not used |
| 26. Derivation of themes | Were themes identified in advance or derived from the data? | Methods, paragraph 3 |
| 27. Software | What software, if applicable, was used to manage the data? | Methods, paragraph 3 |
| 28. Participant checking | Did participants provide feedback on the findings? | Method not used |
| *Reporting* | | |
| 29. Quotations presented | Were participant quotations presented to illustrate the themes/findings? Was each quotation identified? e.g. participant number | Table 2-4 |
| 30. Data and findings consistent | Was there consistency between the data presented and the findings? | Results, Tables 2-4 |
| 31. Clarity of major themes | Were major themes clearly presented in the findings? | Results, Paragraph 1 |
| 32. Clarity of minor themes | Is there a description of diverse cases or discussion of minor themes? | Results, throughout |

Based on Tong A, Sainsbury P, Craig J. Consolidated criteria for reporting qualitative research (COREQ): a 32-item checklist for interviews and focus groups. *Int J Qual Health Care.* 2007;19(6):349-357

© 2020 Butler CR et al. *JAMA Network Open.*

**eTable 2.** Sample Semistructured Interview Guide

| Questions* | Prompt |
|---|---|
| First, can you tell me a little about yourself and your clinical role? | |
| What has it been like for you taking care of patients during the COVID-19 pandemic? | Can you give me some examples of situations that have been particularly challenging? Any situations that have gone well?<br>In what ways have your practices changed? |
| How has the pandemic shaped your clinical practices and how you care for patients? | What was most difficult for you? What worked well?<br>Can you give me some specific examples? |
| Have you encountered any situations in which medical resources were limited or you had difficulty getting patients the treatments they needed during the pandemic? What was that like for you? | Can you give me some examples? |
| Have you talked to patients or family about how their care might be different during the pandemic? What has that been like? | Can you give me some examples?<br>What was that like for you? |
| (For clinicians involved in leadership or triage committee) What has it been like to work with the triage team? | Can you give me some examples?<br>What has been difficult? What has worked well? |
| Have you helped to develop new institutional policies during the pandemic? What has this been like? | Can you give me some examples?<br>What has been difficult? What has worked well? |
| Is there anything else that we have not covered that you would like to bring up? | |

*The interview guide was adapted throughout the study in response to emerging concepts and in order to promote thematic saturation.

© 2020 Butler CR et al. *JAMA Network Open.*
